# Impact of HMGB1, RAGE, and TLR4 in Alzheimer’s Disease (AD): From Risk Factors to Therapeutic Targeting

**DOI:** 10.3390/cells9020383

**Published:** 2020-02-07

**Authors:** Yam Nath Paudel, Efthalia Angelopoulou, Christina Piperi, Iekhsan Othman, Khurram Aamir, Mohd. Farooq Shaikh

**Affiliations:** 1 Neuropharmacology Research Laboratory, Jeffrey Cheah School of Medicine and Health Sciences, Monash University Malaysia, Bandar Sunway, Selangor 46150, Malaysia; iekhsan.othman@monash.edu; 2 Department of Biological Chemistry, Medical School, National and Kapodistrian University of Athens, 11527 Athens, Greece; angelthal@med.uoa.gr; 3 School of Pharmacy, Faculty of Health and Medical Sciences, Taylor’s University, Subang Jaya 47500, Malaysia; khurramaamir@sd.taylors.edu.my

**Keywords:** HMGB1, Alzheimer’s disease, RAGE, TLR4, Neuroinflammation

## Abstract

Alzheimer’s disease (AD) is a devastating neurodegenerative disorder and a leading cause of dementia, with accumulation of amyloid-beta (Aβ) and neurofibrillary tangles (NFTs) as defining pathological features. AD presents a serious global health concern with no cure to date, reflecting the complexity of its pathogenesis. Recent evidence indicates that neuroinflammation serves as the link between amyloid deposition, Tau pathology, and neurodegeneration. The high mobility group box 1 (HMGB1) protein, an initiator and activator of neuroinflammatory responses, has been involved in the pathogenesis of neurodegenerative diseases, including AD. HMGB1 is a typical damage-associated molecular pattern (DAMP) protein that exerts its biological activity mainly through binding to the receptor for advanced glycation end products (RAGE) and toll-like receptor 4 (TLR4). RAGE and TLR4 are key components of the innate immune system that both bind to HMGB1. Targeting of HMGB1, RAGE, and TLR4 in experimental AD models has demonstrated beneficial effects in halting AD progression by suppressing neuroinflammation, reducing Aβ load and production, improving spatial learning, and inhibiting microglial stimulation. Herein, we discuss the contribution of HMGB1 and its receptor signaling in neuroinflammation and AD pathogenesis, providing evidence of its beneficial effects upon therapeutic targeting.

## 1. Introduction

Alzheimer’s disease (AD) is a progressive complex neurodegenerative disorder and an emerging global health concern, afflicting around 50 million worldwide [1]. AD can be described by a steady decline in cognitive function leading to dementia in aging population. The neuropathological hallmarks of AD include acquisition of amyloid-β (Aβ) peptide into the amyloid plaques and intraneuronal neurofibrillary tangles (NFTs), comprising of accumulated Tau protein due to hyper- and/or abnormal phosphorylation [2]. 

AD occurs in two major forms, known as sporadic AD and familial AD. The former is most common (90% of AD cases), affecting people of any age, but mainly above the age of 65 years and it is often referred as late-onset AD (LOAD) [3]. The aetiology of sporadic AD is not well understood, but it has been associated with several genetic, environmental, and lifestyle factors [4]. On the contrary, familial AD is a less prominent form, with an earlier onset. Familial AD has been associated with mutations in three major genes: Aβ precursor protein (*APP)*, presenilin1 (*PSEN1*), and presenilin 2 (*PSEN2*), which induce abnormal overproduction of Aβ [5]. 

Currently, the available AD drugs provide only symptomatic relief without altering the disease progression, thus reflecting the pressing need of effective and safe disease modifying therapies for AD. Most treatment efforts have been focused on modulation of Aβ accumulation mainly through gamma-secretase inhibition, passive vaccination, or amyloid immunotherapy [6], but with poor clinical outcomes [7]. 

The repeated failure of AD clinical trials has shifted drug development towards neuroinflammation, which serves as the link between amyloid deposition, Tau pathology, and neurodegeneration [8]. Among several mediators, high mobility group box 1 (HMGB1) protein has been involved in the initiation and activation of neuroinflammatory responses under pathological conditions. HMGB1 protein is the only family member that has been abundantly and most ubiquitously expressed [9] out of the four proteins (HMGB1, HMGB2, HMGB3, and HMGB4), gaining increased attention in recent time. HMGB1 protein is composed of a chain of 215 amino acids with a molecular weight of 25 kDa, comprising of two DNA binding domains (Box A and Box B) and a negatively charged C-terminal [10,11]. HMGB1 exists in three isoforms (fully reduced HMGB1, sulfonyl HMGB1, and disulfide HMGB1). However, disulfide HMGB1 is the only isoform exhibiting pro-inflammatory cytokine-like activity [12]. The functions of HMGB1 mainly depend on its location, binding partners, and redox states [13,14].

HMGB1 functions as an archetypal alarmin and a typical damage-associated molecular pattern (DAMPs) molecule [15]. Alarmins, being endogenous molecules that can be released into the extracellular settings upon cellular stress or damage, have been shown to activate the immune system [16]. The most well-known alarmins include HMGB1, S100s, heat shock proteins (HSPs), IL-1a, uric acid, cathelicidins, defensins, and thymosins [17]. HMGB1 acts as a chemotactic or pro-inflammatory mediator through direct binding to the receptor for advanced glycation end products (RAGE) and toll-like receptor-4 (TLR4) [11]. RAGE plays a significant role in neurodegeneration, whereas TLR4, being an immune cell receptor, regulates immune response [18]. Both receptors share common signaling pathways to induce inflammation [11] and they have been implicated in the pathogenesis of several diseases with therapeutic targeting potential [12]. 

Accumulative evidence highlights the pathogenic role of HMGB1, RAGE, and TLR4 signaling in AD onset. Upregulation of HMGB1, RAGE, and TLR4 protein levels has been detected in AD peripheral samples [19,20,21]. Moreover, elevated HMGB1 expression was detected in hippocampal neuronal cells of the Aβ_25–35_-induced AD-related model of neuroinflammation and has been correlated with AD progression [18]. In addition, activation of RAGE signaling in AD has been implicated in the production and aggregation of Aβ, NFTs formation, disruption of synaptic transmission, and neuronal degeneration [22]. At the same time, TLR4 activation has been implicated in the induction of neuroinflammation and Aβ deposition [23]. 

Herein, we discuss the pathogenic role of HMGB1 and its principal receptors in AD pathology along with their biomarker potential and the promising clinical outcome of blocking/inhibiting HMGB1, RAGE, and TLR4 in AD experimental studies. 

## 2. The Pivotal Role of Neuroinflammation in AD Onset

Neuroinflammation has emerged as an important feature of AD, presenting a link between accumulation of Aβ and NFTs [24]. It has been associated with AD progression through the activation of astrocytes and microglia, and may present both a driving factor of the disease as well as a response to pathogenic events [25].

There is evidence that inflammation, along with sustained activation of microglia and other immune cells, takes place in AD [24,26,27]. Aβ presence has been shown to induce microglial release of pro-inflammatory cytokines and initiation of APP production, leading to increased Aβ production [28,29]. It is possible that neuroinflammation has an additive effect and serves as a risk factor that increases disease severity by exacerbating Aβ and Tau pathology [24]. Its presence has been associated with several other neurodegenerative dementias, including Parkinson’s disease dementia (PDD), frontotemporal dementia (FTD), and Lewy body dementia (LBD) [30]. Enhanced neuroinflammation due to overexpression of pro-inflammatory cytokines has been previously involved in the elevation of hyperphosphorylated Tau and in the decline of hippocampal function [31]. 

In this context, HMGB1 has been demonstrated to mediate neuroinflammation, and participate in the process of neurodegeneration [32,33]. These data suggest that development of novel pharmacological modulators that can modulate HMGB1 and its receptors (RAGE and TLR4) and control or reduce neuroinflammation may present additional therapeutic strategies to current AD treatment.

## 3. Evidence of HMGB1 Implication in AD Pathogenesis 

HMGB1 is a DNA binding protein localized in the nucleus that translocates to the cytoplasm and eventually is released to the extracellular space during cell activation and apoptosis. Upon stimulation, HMGB1 undergoes post-translational modifications (PTMs) and, based on the redox status of cysteine residues (at positions 23, 45, and 106), it can initiate cytokine production via TLR4 or induce chemotaxis through its interaction with chemokine CXCL12 [34,35]. When released extracellularly, HMGB1 becomes a double-edged sword during neural development and neurodegeneration [33]. 

Recent studies indicate the activation of HMGB1 in AD experimental models. HMGB1 was found localized in the nucleus and the cytoplasm of hippocampal neuron cultures, in Aβ_25–35_-induced AD-related model of neuroinflammation. Upon Aβ_25–35_-treatment, a higher expression of RAGE and TLR4-NF-ĸB (at mRNA and protein level), along with inflammatory mediators (HMGB1, IL-1β, IL-6, and TNF-α), was observed in hippocampal neuronal cells. This finding strongly suggests that neuroinflammation is a crucial contributor of AD and implicates HMGB1 in mediating AD pathogenesis through activation of RAGE/TLR4 signaling, being correlated with AD progression [18]. 

Treatment of mouse microglial (N9) cell line with Aβ (1000 nM) was shown to upregulate HMGB1, IL-1β, and nod-like receptor protein 3 (NLRP3)-inflammasome, indicating a plausible contribution to microglial activation and subsequent inflammation in AD [36]. This is further confirmed by the fact that dipeptidyl vinyl sulfone attenuates Aβ-induced activation of inflammatory process as evident from downregulation of HMGB1, NLRP3, and IL-1β, indicating a reduction of Aβ-induced microglial activation as an emerging approach against AD [36].

In an animal model of early AD monitoring, using 5xFAD transgenic mice, HMGB1 was shown to initiate neurite degeneration with TLR4-myristoylated alanine-rich C-kinase substrate (MARCKS), triggering MARCKS phosphorylation [37]. In fact, HMGB1 initiated neurite degeneration independent of Aβ and the process of Aβ aggregation by disrupting the balance between different Aβ isoforms [37]. HMGB1 was released from necrotic neurons with intracellular Aβ, thus proposing that HMGB1 occurs downstream of the established intracellular Aβ toxicity. The association between HMGB1 and Aβ is therefore bi-directional and HMGB1 may be considered as an independent AD mediator, closely related to the amyloid cascade [37].

The impact of extracellular HMGB1 in the microglial phagocytosis of Aβ40 and Aβ42 has been explored in the perspective of AD [38,39]. A HMGB1 injection with Aβ42 impeded the Aβ42 clearance from the ipsilateral rat hippocampus. Aβ42-induced neurodegeneration was also increased by extracellular HMGB1. Moreover, by inhibiting the microglial phagocytosis, HMGB1 enhanced Aβ mediated neurotoxicity and stabilized the formation of Aβ monomers [38], suggesting its pathogenic role. Therefore, inhibition of extracellular HMGB1 might be a potential therapeutic approach against AD.

A previous study demonstrated the binding affinity between HMGB1 and Aβ40, where the combination of HMGB1 with Aβ and Aβ40 was immunoprecipitated with A-Sepharose-linked antibodies against HMGB1 or Aβ [39]. Mechanistically, extracellular HMGB1 might act as a chaperone for Aβ and reduce the microglial Aβ clearance by interfering with Aβ40 degradation and Aβ42 internalization by microglia. Therefore, extracellular HMGB1 was found to attenuate microglial Aβ clearance and possibly contribute to AD progression [39] by interacting with RAGE and TLR4 that are involved in microglial Aβ phagocytosis [40,41]. 

The p35^−^/^−^/Tg2576 (KO/Tg) mice model of AD with the deletion of p35 (a neuronal activator of CDK5) exhibited synaptic dysfunction and increased neuronal cell death, which is correlated with activated microglial infiltration and upregulated HMGB1 expression. Importantly, microglial infiltration in the dentate gyrus (DG) and CA1 led to increased HMGB1 secretion, which might increase neuronal apoptosis when combined with Aβ [42]. Therefore, blocking HMGB1 expression may provide protection against neuronal cell death.

Despite several studies on adult hippocampal neurogenesis (AHN) in AD [43,44], no similar conclusion or mechanisms of regulation have been identified [45]. This is evident of the controversial studies that showed AD to downregulate hippocampal neurogenesis with progression of disease [46] or to increase hippocampal neurogenesis [47]. Nevertheless, disruption of AHN at early stages might mediate the AD pathogenesis, indicating a therapeutic approach towards its prevention and treatment [48]. 

In this regard, HMGB1 has emerged as an inducer of differentiation of neural progenitor cells (NPCs). A study using TgCRND8 mice (an animal model of FAD) demonstrated no significant growth of new mature neurons in the hippocampi when compared to WT mice, indicating decreased survival and/or integration of newborn neurons. Of importance, HMGB1 and Aβ_1–42_ activated a potential reparative mechanism by promoting neuronal differentiation of adult hippocampal NPCs via the activation of the RAGE/NF-κB cascade [49], thus demonstrating the pro-neurogenic potential of HMGB1. It is therefore evident that HMGB1 activation in AD occurs through the interaction with Aβ and that it is not only a risk factor but may also exert a restorative effect in AHN.

Disruption of learning and memory represents important hallmarks of AD and intracerebroventricular (ICV) injection of HMGB1 (10 μg) in WT, TLR4^−/−^, and RAGE^−/−^ mice was shown to block these functions. The HMGB1 injection was found to affect memory encoding, as demonstrated by reduced novel object preference index in novel object recognition test (NORT). The HMGB1 amnesic effect was mediated by RAGE and TLR4, as evident by the blockade of memory impairment upon injection of TLR4 antagonist in RAGE-deficient mice [50].

## 4. Implication of RAGE in AD 

RAGE belongs to the immunoglobulin (Ig) superfamily [51] and is widely expressed on several cell types, ranging from vascular cells (endothelial and smooth muscle cells) to immune/inflammatory cells (neutrophils, monocytes/macrophages, lymphocytes, and dendritic cells) [52,53,54,55]. RAGE binds to several DAMPs (including AGEs, HMGB1, S100s, and DNA) and mediates differential cellular responses, being a crucial regulator of the innate immune response. Due to its ability to identify a range of structurally unrelated endogenous and exogenous ligands, RAGE is regarded as a pattern recognition receptor (PRR) [56]. The PRRs comprise of TLRs, nucleotide-binding oligomerization-like receptors, and several other DNA sensors. 

There is evidence that RAGE signaling is implicated in an array of inflammatory [57] and neurodegenerative diseases [58], including AD [22,59]. Amyloid plaques in AD develop due to the overproduction of Aβ and/or the failure of Aβ clearance, promoting its deposition. Of importance, RAGE has been demonstrated to play a crucial role in Aβ production and at the failure of Aβ clearance [22]. RAGE, due to its molecular structure and nature, facilitates circulating plasma Aβ entry into the brain through BBB [60]. Along with its decoy receptor soluble RAGE (sRAGE), they may confer protection against AD pathogenesis by manipulating the transport of Aβ into the brain or by modulating the inflammatory mechanisms [61]. sRAGE, an isoform of RAGE lacking the transmembrane domain, competes with the cell-surface RAGE for ligand binding, and has been shown to be involved in the removal or neutralization of circulating ligands, acting as a decoy molecule [61].

RAGE activation induces Aβ production and the aberrant hyperphosphorylation of Tau. It can activate microglia and astrocytes in a reactive as well as in an inflammatory state, thus aggravating AD pathogenesis by inducing a cycle of inflammation and cellular stress [62]. In a cross-sectional clinical study of different dementia patient types, the levels of AGEs and its receptor RAGE were found upregulated in AD, whereas the levels of sRAGE were decreased, indicating that AGE, RAGE, and sRAGE might contribute to AD pathology [63]. 

In a clinical study of AD and non-demented (ND) patients, the upregulation of RAGE levels in the hippocampus and inferior frontal cortex in post-mortem AD patients was correlated with the severity of brain pathology. Increased immunoreactivity of RAGE has been observed mainly in neurons, microglia, and astrocytes in the affected areas of AD patients [40]. An earlier clinical study unraveled that microvascular RAGE levels were increased with AD onset and were upregulated consecutively in relation to AD severity. In concert, a significant upregulation in endothelial RAGE immunoreactivity was observed in severe Braak V-VI AD patients compared to aged-controls, as well as in patients with early AD pathology. Similarly, a notable elevation in endothelial RAGE immunoreactivity was observed in patients exhibiting early AD-like symptoms compared to aged controls with no reported AD pathology [64].

RAGE has also been demonstrated as a modulating cofactor of the Aβ impact on neuronal function. An experimental study investigating the effects of RAGE in an Aβ-rich environment employed a transgenic (Tg) mouse model with targeted neuronal overexpression of RAGE and mutant APP (mAPP). Double Tg mice (mutant APP/RAGE) exhibited early spatial learning and memory impairments, supplemented by an altered activation of synaptic plasticity markers (CREB and MAPK) and amplified neuropathologic findings, before the detection of these alterations in mAPP mice [65]. On the contrary, when Tg mice with a dominant-negative RAGE construct targeted to neurons were crossed with mAPP animals, protection of spatial learning/memory deficits and reduced neuropathological changes were observed. These findings suggest that RAGE acts as a cofactor for Aβ-mediated neuronal perturbation in experimental models, with a potential as a therapeutic target to improve cellular disruption [65]. 

Similarly, Tg mice expressing mAPP in neurons and RAGE in microglia showed increased production of IL-1β and TNF-α, enhanced infiltration of microglia and astrocytes, Aβ aggregation, decreased AChE activity, and rapid disruption of spatial learning/memory [66]. RAGE-facilitated generation of pro-inflammatory mediators enhanced accumulation of Aβ via a positive feedback loop, activating the RAGE receptor that will ultimately exacerbate neuroinflammation and amyloid pathology [66]. Earlier studies investigating mAPP mice with genetic deletion of RAGE (mAPP/RO) have shed more light on the impact of RAGE on accumulation of Aβ, amyloid pathology, learning and memory impairments, indicating that an additional underlying mechanism is a part of its role in the cleavage of APP to release Aβ [67]. The cytosolic domain of neuronal RAGE was implicated in the abnormal APP processing and Aβ production via the enhancement of β- and γ-secretase activity. The deletion of RAGE was found to block the initiation of GSK3β and p38 MAPK signaling axis in the Aβ milieu of mAPP mice. Furthermore, RAGE deficiency conferred a defensive effect on the learning and memory deficits in mice overexpressing mutated APP [67]. These findings further support the benefits of targeting RAGE to block the aberrant APP-Aβ metabolism and hinder AD progression.

Overall, there are several RAGE-related signaling axes including the RAGE/Ca^2+^/calmodulin-dependent protein kinase kinase-β (CaMKK-β)-AMPK, the RAGE/extracellular signal regulated kinase 1/2 (ERK1/2), RAGE/GSK-3β, and RAGE/NF-κB that have been implicated in AD, which are all associated with the regulation of abnormal Tau hyperphosphorylation and Aβ pathology [22].

## 5. TLR4 Involvement in AD Pathogenesis

TLRs belong to the family of microbe-sensing receptors and contribute to innate immune defense against infection through binding to microbial molecules [68]. To date, 10 TLRs located either at the cellular surface (TLR1, TLR2, TLR 4, TLR5, TLR6, and TLR10) or in the endosome (TLR3, TLR7, TLR8, and TLR 9) have been reported [69]. TLR4 is a transmembrane protein that belongs to the PRRs family [70], which is widely explored in the context of AD pathology. The binding of pathogen-associated molecular patterns (PAMPs) to TLR4 activates the NF-κB signaling axis, resulting in the synthesis and secretion of inflammatory cytokines [71]. Due to the known contribution of the innate immune system in AD, TLR4 has received increased attention and has been extensively studied in AD [72]. TLR4 is also instrumental in driving the binding of fibrillary amyloid and its phagocytosis by microglia in AD [73]. The presence of amyloid is detrimental in activating the TLR4-mediated NF-κB/MAPK inflammatory axis, promoting the discharge of pro-inflammatory and neurotoxic cytokines (IL-1 β, IL-6, and TNF-α) [23,74]. Importantly, microglial activation by Aβ has been shown to require a functional receptor complex of TLR4, MD-2, and CD14 [23].

Additionally, loss-of-function mutations of the *TLR4* gene have been associated with inhibition of microglial and monocytic activation by accumulated amyloid peptide, leading to a decreased expression of the inflammatory markers (IL-6 and TNF-α) and nitric oxide, implicating TLR4 in the neuroinflammation of AD. Moreover, TLR4 mRNA was found elevated in *APP*-overexpressing mice. TLR4 was also shown to mediate Aβ-induced microglial neurotoxicity, as well as Aβ-mediated activation of murine microglia and human monocytes [23].

Despite the fact that innate immune/inflammatory responses contribute to the AD pathology, the underlying mechanism is not completely understood. In a study investigating the contribution of TLR4 in Aβ-induced upregulation of cytokines and chemokines, Aβ-induced microglial and astrocytes stimulation, and migration of leukocytes, there was an upregulation of TNF-α, IL-1β, IL-10, and IL-17 levels in the brain of TLR4 WT AD mice. However, elevation of these cytokines was not reported in *TLR4*-mutant AD mice as compared to the *TLR4*-mutant non-transgenic littermates. In addition, the expression levels of the microglia marker CD11b and the reactive astrocyte marker GFAP were upregulated in the brain of *TLR4*-mutant AD mice compared to *TLR4*-WT AD mice, without difference at the levels of the common leukocyte antigen CD45. This TLR4-dependent upregulation of cytokines in the AD mouse model indicates the involvement of TLR4 signaling in disease progression and its potential therapeutic targeting [75]. 

The triggering receptor expressed on myeloid cells 2 (TREM2) protein, a crucial innate immune receptor in the brain, behaves as a protective mechanism against AD where TLRs play a significant role. TREM2 overexpression was associated with upregulation of the cellular activity of Aβ_1–42_, and promoted its clearance by BV-2 cells, while it decreased the expression of inflammatory markers (IL-1β, IL-6, and TNF-α). It also contributed to decreased expression of other members of TLR family in BV-2 cells, such as TLR4, TLR2, and TLR6 [76]. Overall, TREM2 was shown to attenuate Aβ_1–42_-mediated neuroinflammation in BV-2 cells via downregulation of TLR signaling pathway. TLR4-driven inflammation was further negatively controlled by TREM2 [77]. Lipopolysaccharide (LPS) injection into the APP/PS1 transgenic AD model mimics systemic inflammation in the development of AD whereby TLR4 expression was upregulated. On the contrary, expression of TREM2 was markedly decreased in APP/PS1 mice, reflecting that the negative modulatory effect of TREM2 on inflammation could be inhibited by LPS-induced hyperactive TLR4. Thus, an imbalance of TLR4/TREM2 may present a potential link between AD and systemic inflammation [78].

In a clinical study of post-mortem human brains, an upregulation of the TLR4, IL-6, and TNF-α mRNA levels was observed at the frontal cortex of AD subjects as compared to age-matched controls [21]. Similarly, in a mouse model of hippocampal differentiation (at 7 days post-lesion) without amyloidosis (i.e., the entorhinal cortex lesioned mouse), hippocampal TLR4 and IL-1β mRNA expression levels were significantly elevated compared to sham-lesioned mice. However, during reinnervation phase (at 21 days post-lesion) there was no significant difference at the TLR4, IL-1β, IL-6, and TNF-α mRNA levels compared to sham-lesioned mice [21]. This finding suggests that the contribution of TLR4 in neuroinflammatory process during AD is not only triggered by amyloidosis, but also by an amyloid independent differentiation process that occurs in the early phases of the disease [21].

A study investigating the role of TLR4 signaling and microglial activation in early stages of AD pathology reported that a non-functional mutation in the TLR4 gene reduced Aβ-induced activation of microglia in the AD mice model at 5 months of age, when the brain deposits of Aβ usually increase. In fact, no difference was noted in the cerebral Aβ deposits and buffer-soluble Aβ amounts between TLR4 wild-type (TLR4W Tg) and *TLR4* mutant AD (TLR4M Tg) mice at the early stages of β-amyloidosis [79]. This finding indicates that TLR4 signaling does not alter the production of Aβ and the onset of Aβ deposition. On the contrary, the 9-month-old TLR4M Tg mice exhibited an elevation in the quantity of cerebral Aβ deposits and soluble Aβ42, associated with special learning impairment and decreased CCL3 expression, suggesting that microglial activation via TLR4 could be neuroprotective [79].

Furthermore, the TLR signaling axis contributes to the clearance of Aβ-deposits in the AD brain. The contribution of TLR4 in amyloidogenesis has been revealed in vivo. The Mo/Hu APPswe PS1dE9 mice, which are homozygous for a destructive mutation of TLR4 (Tlr^Lps-d^/Tlr^Lps-d^), showed increased diffuse and fibrillar Aβ deposits compared to TLR4-WT mouse models [41], indicating that manipulation of the innate immune responses via the TLR4 axis may decrease Aβ load and cell injuries in AD brain.

LPS was shown to activate a greater number of microglia in the young TgAPP/PS1 mice (without Aβ deposition) compared to young WT mice, whereas its ability to activate microglia in old TgAPP/PS1 mice is less prominent (with Aβ deposition) as compared to old WT mice. TLR4 signaling is disrupted in TgAPP/PS1 mice, explaining the remarkable contrast in TLR4 signaling activation between WT and TgAPP/PS1 mice, as well as before and after Aβ deposition in the brain [80]. Hence, microglial TLR4 signaling is inhibited in the AD mouse model, indicating that dysregulated TLR4 signaling may be associated with Aβ accumulation in the brain [80]. 

The relationship between neuroinflammation, autophagic activity, and TLR4 stimulation has also been investigated in Tau transgenic AD mice. TLR4 stimulation through LPS injection triggers microglial/macrophage inflammatory activation, further enhancing the autophagic flux in the mouse brain. Moreover, chronic mild TLR4 stimulation improves AD-related pathology, as well as synaptic impairments, in Tau-transgenic mice [81].

Activation of TLR signaling can further aggravate AD via initiation of the inflammatory process, Aβ deposition, and oxidative stress [82]. TLR4 is not only essential for regulation of the inflammatory process, but also for the uptake as well as the phagocytic elimination of Aβ plaques [41]. TLR4 activates the phagocytosis of Aβ peptides [73,83], as well as contributes to the formation of Aβ plaque [84,85].

Taken all together, it is evident that modulation of TLR4 signaling pathways could exert a significant impact on AD pathology, mainly by changing the inflammatory state of microglia/macrophages [86].

## 6. HMGB1, RAGE, and TLR4 as Potential Clinical Biomarkers of AD

AD is a multifactorial disease that develops gradually with symptoms progressing with time, reflecting the need for early intervention [87]. In this regard, exploring biomarkers in AD that can predict the disease and monitor its progression while providing insight into the outcome of therapy are needed. The cerebrospinal fluid (CSF) levels of Aβ, fragments, and p-Tau or total-Tau are extensively used biomarkers for AD [88,89], but their diagnostic accuracy varies between different centers [90]. Furthermore, there is a growing interest in exploring biomarkers of AD that relate to neurodegeneration and BBB dysfunction [91]. This section focuses on novel potential AD biomarkers which are well implicated in AD pathology, such as HMGB1 and its principal receptors (RAGE and TLR4).

A clinical study validating the non-invasive clinical biomarkers of BBB dysfunction and neuroinflammation to evaluate the progression towards neurodegeneration in mild cognitive impairment (MCI) and AD patients detected upregulated expression and/or release of serum HMGB1 and sRAGE, correlated with Aβ levels in AD patients [92]. Interestingly, the elevation of serum HMGB1 levels was observed in patients with MCI as compared to controls or AD patients. Moreover, soluble thrombomodulin (sTM) antigen (a marker of BBB disruption) activity was significantly upregulated in MCI and AD patients. These findings suggest that HMGB1 and sRAGE may act as clinical biomarkers for AD progression [92]. A similar type of upregulation was also reported in the brain tissues of AD patients, suggesting that HMGB1 might accumulate in either extracellular or intracellular regions [19]. Detection of HMGB1 on Aβ40 plaques in AD brains using a specific anti-Aβ40 antibody demonstrated that HMGB1 accumulates extracellularly on Aβ plaques containing Aβ40 in AD brains [39]. A similar type of HMGB1 immunoreactivity was noted in the senile plaques, where levels of HMGB1 protein were found upregulated in AD brains [38]. Evaluation of the HMGB1 concentrations in the CSF of human AD patients showed that HMGB1 levels were unchanged in healthy controls and FTLD patients. However, in a group of AD patients, upregulation of HMGB1 levels was associated with a rapid progression of dementia, further suggesting that CSF levels of HMGB1 might represent a marker of neurodegenerative progression [37]. 

In a clinical study, RAGE was found co-localized near neuritic plaque deposits in the cells of Aβ-comprising blood vessels, and in endothelial, neuronal, and microglial cells in AD brain tissue at much higher concentrations compared to age-matched control-derived tissues [93]. In plasma samples of AD patients, the affinity-purified IgGs binding to fragment of RAGE were elevated by three-fold, whereas RAGE, IgG, and Aβ titers were negatively correlated with cognitive status compared to control samples. However, individuals with severe cognitive impairment tend to demonstrate higher IgG titers [94]. These data suggest that the measurement of specific Aβ and RAGE IgGs and/or their protein complex might represent a confirmatory test for AD or AD susceptibility [94]. 

Increased RAGE expression was observed in the capillaries of the AD brain as compared to controls. The significant negative correlations obtained between the Aβ burden of amyloid plaques and RAGE-positive capillaries in AD brains suggest that RAGE is a crucial factor that influences Aβ burden [95]. In an effort to elucidate the AD-related alterations in BBB-associated Aβ receptors, RAGE immunoreactivity was detected in neurons from control hippocampi, whereas a significant reduction in neuronal RAGE immunoreactivity was observed in AD cases. However, a higher concentration of RAGE was detected in AD hippocampi as compared to controls by Western immunoblotting. These observations suggest that AD is linked with changes in the relative distribution of RAGE in the human hippocampus [96].

Significant elevation in the expression of RAGE levels was also observed in post-mortem AD patients (hippocampus and inferior frontal cortex) where the increased RAGE expressions were positively correlated with the severity of brain pathology [40]. Decreased expression levels of sRAGE, which inhibits RAGE signaling, have been reported in the plasma of patients with AD when compared to those with vascular dementia or normal controls [97].

A clinical study conducted in Northern Han Chinese populations demonstrated an increased plasma level of TLR4 in the peripheral blood mononuclear cells (PBMCs) along with elevated TLR4 mRNA and protein levels in LOAD patients compared to healthy controls [98]. On the contrary, a downregulation of TLR4 protein expression was observed in plasma/serum of AD patients who had more Aβ plaques than in patients with other dementia-related diseases [99]. A population study in northern Italy including 626 AD patients also reported that the +896A TLR4 pro-inflammatory allele was overrepresented in AD patients, suggesting that TLR4 single nucleotide polymorphism (SNP) may be a genetic marker of AD susceptibility [100].

## 7. HMGB1, RAGE, and TLR4 Inhibition/Blockade as a Potential Therapy against AD

Based on the evidence that HMGB1, RAGE, and TLR4 contribute to the pathogenesis of AD, their targeting might be instrumental in elucidating the plausible underlying mechanism associated with AD pathogenesis. Up to date, several HMGB1, RAGE, and TLR4 blocking/inhibiting strategies have demonstrated promising outcomes against AD on experimental studies and are discussed below.

### 7.1. Effects of HMGB1 Neutralization in AD

The available therapeutic strategies to block/inhibit extracellular HMGB1 include anti-HMGB1 monoclonal antibody (mAb), specific HMGB1 inhibitors (glycyrrhizin and its derivatives), and HMGB1 interference (shRNA) in AD-like experimental settings.

The therapeutic use of anti-HMGB1 antibodies against several HMGB1-mediated pathologies was mainly focused on elucidating the involvement of HMGB1 in several pathological conditions, validation of protein targets, and the efficacy of potential therapeutic approaches [101]. 

The therapeutic potential of anti-HMGB1 mAb has been recently reviewed in several HMGB1-mediated diseases including PD, epilepsy, TBI, and AD [102]. The anti-HMGB1 mAb treatment in 5xFAD mice ameliorated cognitive impairment at a similar level to WT mice. Administration of the anti-HMGB1 mAb during 1-6 and 3-6 months of age significantly decreased the DNA damage in the cerebral cortex of 5xFAD mice to the normal levels (at 6 months). However, treatment with anti-HMGB1 mAb did not modulate the expression of human APP in the brains of 5xFAD mice but inhibited the HMGB1-induced elevation of Aβ monomers and oligomers [37]. In addition, anti-HMGB1 mAb treatment increased the microglia-specific marker, Iba1, mainly around Aβ aggregates, and enhanced phagocytosis of the Aβ-HMGB1 complex. Anti-HMGB1 mAb further blocked HMGB1 activity with TLR4, inhibited phosphorylation of MARCKS at Ser46, and prevented neurite degeneration, indicating its beneficial effects in modifying disease progression [37]. 

Glycyrrhizin is a small-molecule inhibitor of extracellular HMGB1 cytokine activity [103] with the potential to penetrate BBB [104]. The neuroprotective effect of glycyrrhizin has been demonstrated in an array of neurological disorders, including epilepsy [105], TBI [106], and PD [107]. Although the therapeutic effect of glycyrrhizin has not been demonstrated in AD mice with developed Aβ pathology, its effects have been extensively evaluated against several AD-like pathologies such as LPS-induced neuroinflammation and cognitive deficits, as well as in surgery-induced cognitive decline. Glycyrrhizin and its derivatives have exerted promising effects mainly by inhibiting HMGB1, improving memory deficits, and reducing the levels of inflammatory cytokines [108,109,110]. The experimental studies with promising outcomes upon HMGB1 inhibition in AD-like pathologies are summarized in Table 1.

The complexity of HMGB1 inhibition/blockade is attributed to the existence of three different isoforms of HMGB1 with distinct and different functions [111]. Among the three HMGB1 isoforms, disulphide-HMGB1 is the only isoform possessing pro-inflammatory cytokine-like activity that activates macrophages/monocytes and other cells to produce cytokines, as well as inflammatory mediators [14]. Of importance, therapeutic targeting of HMGB1 in AD might be immature at present since it is implicated at multiple levels in the regulation of immune response, and the precise underlying mechanism of its involvement in AD has not been completely understood. Despite this limiting aspect, the encouraging data of HMGB1 neutralization mAb, glycyrrhizin, and its derivatives in AD-like pathologies suggest that it may present a promising target and needs further investigation [112].

### 7.2. Effects of RAGE Inhibition in AD

Several pre-clinical and clinical studies have reported the beneficial effects of RAGE inhibitors in AD-like conditions. Genetic blockade of RAGE in mAPP (mAPP/RO) mice exhibited decreased cerebral amyloid pathology with suppressed abnormal APP-Aβ metabolism by downregulating activity of β- and γ-secretase. It also ameliorated learning and memory impairment as compared to mAPP mice [67]. Furthermore, mAPP mice deficient to RAGE (mAPP/DN-RAGE) demonstrated reduced production of Aβ_40_ and Aβ_42_ and lowered activity of β- and γ-secretase as compared to mAPP mice. RAGE-deleted mAPP brain exhibited inhibition of p38 MAP kinase and GSK3β activity. These findings indicate the therapeutic potential of RAGE targeting based on its ability to inhibit APP-Aβ metabolism and hinder the progression of AD [67]. Inhibition of neuronal RAGE could demonstrate cytoprotective effects by preserving neuronal function at early stages of the disease. This is further supported by studies of transgenic mice with a dominant-negative RAGE construct targeted to neurons crossed with mAPP animals which exhibited protection of spatial learning/memory deficits and reduced neuropathological alterations, reflecting that RAGE can improve cellular dysfunction [65].

Moreover, several HMGB1 inhibition approaches, including TTP488, sRAGE-mesenchymal stem cells (MSCs), FPS-ZM1, matrine, pentamidine, hesperidin, and linguizhugan, have revealed promising outcomes in experimental AD models mainly by inhibiting RAGE expression, decreasing production of Aβ, reducing Aβ deposition, oxidative stress, and inflammatory cytokines, while improving spatial learning and memory (Table 2) [113,114,115,116,117,118].

Although pre-clinical studies of RAGE inhibition in AD strengthen the fact that RAGE might represent a potential therapeutic target, future pre-clinical and clinical studies are warranted to evaluate the safety and therapeutic efficacy of RAGE antagonists against AD [22].

### 7.3. Effects of TLR4 Blockade in AD

Aβ-mediated TLR4 activation actively promotes neuroinflammation in AD, suggesting that blockade/inhibition of TLR4 activation may suppress neuroinflammatory processes [75]. 

In an experimental study of immunological preconditioning with TLR4 agonist LPS, Monophosphoryl lipid A (MPL) was shown to upregulate IFN-β-positive cells and reduce hippocampal TNF-α-positive cells of Aβ-treated rats. Reduction in TNF-α could be the result of increased IFN-β levels via the TLR4 signaling axis [119]. The release of IFN-β upon pre-treatment with TLR4 agonists might be a promising neuroprotective strategy against neurodegeneration in AD. Spontaneous loss-of-function mutation in the TLR4 gene suppressed activation of microglia and monocytes by aggregated Alzheimer’s amyloid peptide, leading to the downregulation of inflammatory markers IL-6, TNF-α, and NO [23]. TLR4 stimulation with detoxified ligand MPL ameliorated AD-like pathology in APPswe/PS1 mice, as evident by the reduced number and size of Aβ deposits, as well as by the quantity of soluble Aβ in the brain [120].

A wide range of therapeutic compounds (Table 3) have demonstrated their efficacy in animal models of AD-like pathologies, mainly by inhibiting TLR4 expression, suppressing microglial activation and pro-inflammatory cytokine levels, ameliorating learning and memory functions, inhibiting oxidative stress, and reducing apoptotic cell death and Aβ load (number and size of Aβ deposit) [120,121,122,123,124,125,126].

These findings indicate that therapeutic targeting of TLR4 may present a promising therapeutic approach for the symptomatic improvement and slowing of AD progression. 

## 8. Discussion and Future Implications

AD is the major cause of dementia worldwide, accounting for 50–70% of all cases [127]. AD is considered as a complex disorder, with multiple molecules and several contributing factors playing a significant role [128]. At the early stages of AD, the immune system contributes to the elimination of amyloid peptides. However, with disease progression, inability to clear toxic Aβ peptides along with an activation of the innate immune system might lead to the initiation of chronic inflammatory phenomena in the brain [129,130]. To date, there is lack of disease-modifying therapy against AD despite the tremendous research efforts, reflecting the increased complexity of the disease. There is an intensive need to explore novel therapeutic strategies that will prevent AD and/or retard the disease progression. However, due to the lack of precise understanding about the mechanisms underlying AD pathogenesis, the development of treatment strategies against AD is complex, which is evident by the repeated failure of drugs in the clinical trial. 

**Figure 1:**
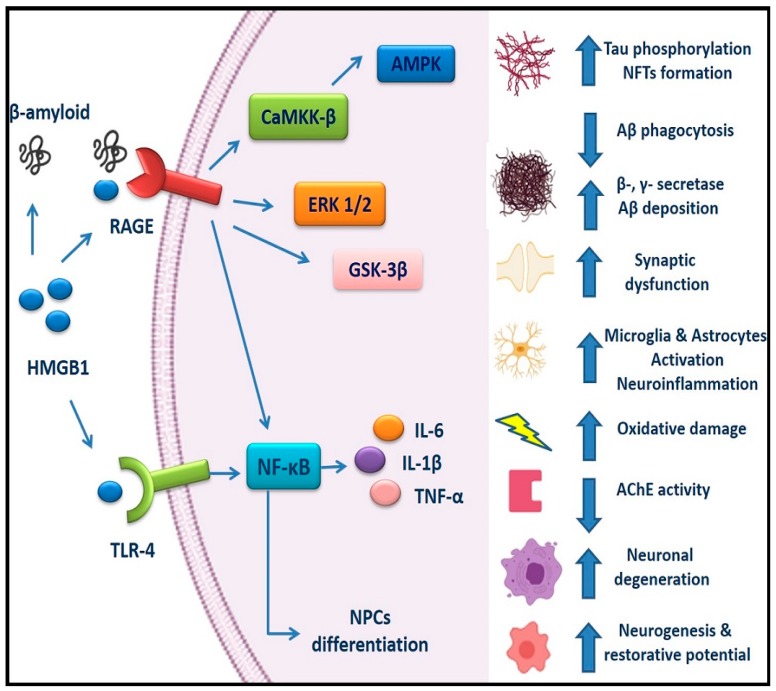
HMGB1/RAGE and HMGB1/TLR4 signaling pathways in AD: HMGB1 can interact with extracellular Aβ peptides and decrease Aβ deposition by inhibiting Aβ clearance by microglia, as well as increasing β- and γ-secretase activity. RAGE enhances production of Aβ, abnormal Tau hyperphosphorylation, and NFTs formation. HMGB1/RAGE and HMGB1/TLR4 signaling induce neuroinflammation by activating the NF-κB pathway, increasing production of pro-inflammatory cytokines including TNF-α, IL-6, and IL-1β, activating microglia and astrocytes in a reactive and inflammatory state, and thus aggravating the AD pathogenesis through a vicious cycle of inflammation and oxidative damage. RAGE/CaMKK-β-AMPK, the RAGE/ERK1/2, RAGE/GSK-3β, and RAGE/NF-κB pathways have been involved in the regulation of abnormal Tau hyperphosphorylation and Aβ pathology. RAGE signaling has been also implicated in synaptic dysfunction, reduced AChE activity, and neurodegeneration. However, activation of RAGE/NF-κB pathway by HMGB1 in adult NPCs promotes neuronal differentiation and formation of new neurons, leading to increased adult neurogenesis. In addition, HMGB1 may play dual roles in AD pathogenesis, since it can also contribute to reparative mechanisms in the AD brain. AD, Alzheimer’s disease; HMGB1, High mobility group box 1; RAGE, Receptor for advanced glycation end products; TLR4, Toll-like receptor 4; Aβ, Amyloid beta; NFTs, Neurofibrillary tangles; CaMKK-β,Ca^2+^/calmodulin-dependent protein kinase kinase-β; ERK1/2, Extracellular signal regulated kinase ½; NPCs, Neural progenitor cells; IL, Interleukin; NF-κβ, Nuclear factor κ light chain enhancer of activated β cells; TNF-α, Tumor necrosis factor-α.

Intervention at the early pathological stages of AD is considered of primary importance and HMGB1 with its receptors (RAGE and TLR4) has gained increased attention in AD pathogenesis (Figure 1). Upregulation of HMGB1, RAGE, and TLR4 levels in AD patients [92,95,98] and experimental models [18] indicate their involvement in the disease pathogenesis, presenting a possible risk factor and a therapeutic target.

**Figure 2:**
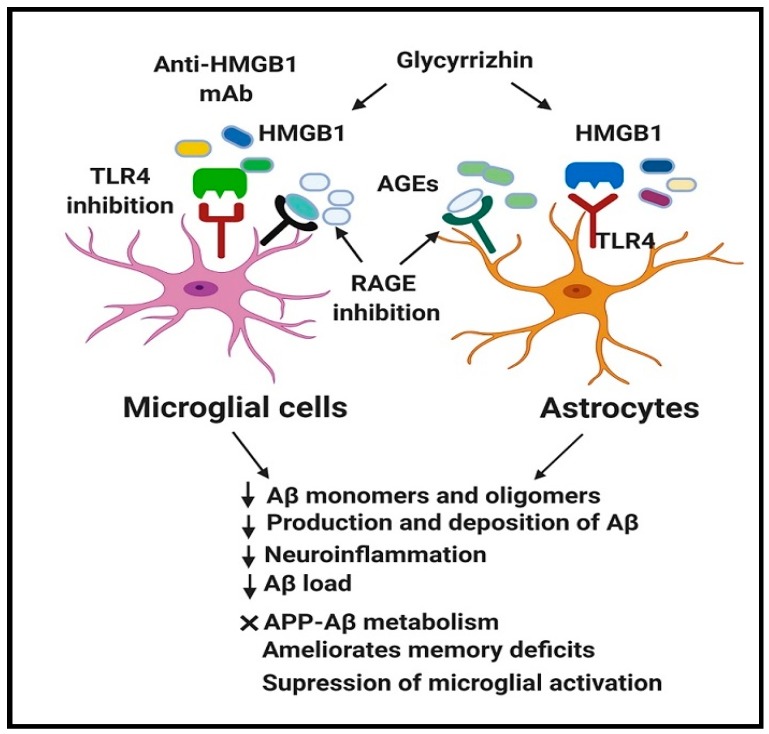
Beneficial effect of HMGB1, RAGE, and TLR4 inhibition in AD.

Interestingly, inhibition/blockade of HMGB1, RAGE, and TLR4 in experimental AD-like pathologies demonstrated promising outcomes by modifying AD progression through suppression of neuroinflammation, reduction of Aβ load (number and size of Aβ deposit) and Aβ production, improvement of spatial learning and inhibition of microglial activation (Figure 2) (Table 1, Table 2 and Table 3).

Therapeutic blockade of HMGB1, RAGE, and TLR4 in AD using their respective inhibitors demonstrated promising outcomes in modifying AD progression through inhibition of HMGB1, RAGE, and TLR4 expression. Moreover, neutralization of HMGB1, RAGE, and TLR4 leads to the decrease in Aβ monomers and oligomers, suppression of abnormal APP-Aβ metabolism, inhibition of neuroinflammation, reduction of Aβ load (number and size of Aβ deposit) and Aβ production, amelioration of spatial learning and memory deficits, and suppression of microglial activation. These reflect that HMGB1, RAGE, and TLR4 might represent promising therapeutic targets against AD. AD, Alzheimer’s disease; HMGB1, High mobility group box 1; RAGE, Receptor for advanced glycation end products; TLR4, Toll-like receptor 4; Aβ, Amyloid beta; APP, Amyloid precursor protein; AGEs, Advanced glycation end products.

However, on a broader aspect, we should acknowledge the fact that developing treatment strategies for AD is much complex. Also, it is worth noting that due to the multifactorial, heterogeneous, progressive, and interactive pathophysiology of AD, there is a need for personalized combinatorial treatment that differs based on patients’ medical history and disease stages [131].

The encouraging outcome of HMGB1, RAGE, and TLR4 blockade/inhibition indicates that extensive research is highly demanded to elucidate their pathogenic role in AD, along with future clinical studies to validate their therapeutic potential.

## Figures and Tables

**Table 1:** Summaries of studies reporting HMGB1 targeted therapies in AD and related pathology.

S.N.	Interventions	Model	Treatment Schedule	Observations	References
1	HMGB1 short hairpin RNA (shRNA)	Aβ_25–35_-induced (25 μmol/L) neuroinflammation in hippocampal neuron cultures	Pre-treated for 24 h	HMGB1 shRNA inhibits nuclei to cytoplasmic translocation of HMGB1 after treatment with Aβ_25–35._ HMGB1 shRNA inhibits NF-ĸB activity, reduced RAGE and TLR4 expression and inhibited inflammatory cytokine (HMGB1, IL-1β, IL-6, and TNF-α) secretion after Aβ_25–35_ treatment.	[18]
2	Anti-HMGB1 mAb (1 mg/kg, S.C. injection) (1 injection/week)	5xFAD transgenic mice overexpressing the mutant human APP	Administered for 1–6 months or 3–6 months	Anti-HMGB1 mAb treatment decreases the amount of Aβ aggregates and the oligomers as well as enhance Aβ phagocytosis by microglia. Treatment with Anti-HMGB1 mAb inhibited the degeneration of neurite even in the presence of Aβ plaques and completely ameliorated the cognitive dysfunction.	[37]
3	Glycyrrhizic acid (GA) (50 and 100 mg/kg, I.P.)	LPS (250 μg/kg) -induced neuroinflammation and cognitive impairment in the C57 mice (4–5 weeks old)	Once daily for 1 week	GA treatment ameliorate LPS-induced cognitive decline and neuronal damage by decreasing the escaped latency in MWM test and upregulating the number of Nissl-stained cells and normal neurons in the hippocampus respectively. Treatment with GA reduces LPS-induced neuroinflammatory response in cortex and hippocampus (TNF-α and IL-1β).	[109]
4	Glycyrrhizin (GL) (16.8 mg/kg, I.P.)	p35^-^/^-^/Tg2576 mice (p35 deletion in Tg2576 mice)	Every alternate day for 1 week	GL treatment reduced neuronal cell death	[42]
5	GL (30 mg/kg, orally)	Surgery induced cognitive decline in C57BL/6 mice	Once daily for 3 days pre-operatively	GL pre-treatment reduces splenectomy surgery-induced neuroinflammation (TNF-α, IL-6 and IL-1β). Pre-treatment with GL attenuates the increases of Hippocampal Aβ levels, Tau phosphorylation and HMGB1 upregulation induced splenectomy surgery. GL rescued the splenectomy surgery induced spatial memory deficits as demonstrated by the shorter swimming latency as well as distance in MWM test.	[110]
6	GL (30 and 50 mg/kg, orally)	LPS (3 mg/kg, I.P.)-induced neuroinflammation and cognitive impairment in the C57BL/6 mice	Once a day for 3 days prior to LPS injection	GL ameliorated the LPS-induced memory deficit as evident by prolonged swimming time in MWM trial. GL administration reduced the markers of inflammation (TNF-α and IL-1β mRNA) and protein expression of COX-2 and iNOS.	[108]
7	18α-glycyrrhetinic acid (GA) (20 μg/mL)	AD nematode models (WT *Caenorhabditis elegans)*	-	Administration of 18α-GA increased the levels of proteasome activities leading to a skinhead-1 and proteasome activation-dependent life span extension. 18α-GA treatment reduces Aβ toxicity and reduces Aβ-induced neuronal cell death.	[132]

HMGB1, High mobility group box 1; GL, Glycyrrhizin; GA, Glycyrrhizic acid; AD, Alzheimer’s disease AD; RAGE, Receptor for advanced glycation end products; TLR4, Toll-like receptor 4; Aβ, Amyloid beta; APP, Amyloid precursor protein; WT, Wild-type; FAD, Familial AD; IL, Interleukin; IBA1, ionized calcium-binding adapter molecule 1; iNOS, Inducible nitric oxide synthase; COX-2, Cyclooxygenase-2; NF-κB, Nuclear factor κ light chain enhancer of activated B cells; TNF-α, Tumor necrosis factor-α, LPS, Lipopolysaccharide; MWM, Morris water maze; SC, Subcutaneous.

**Table 2:** Summaries of studies reporting RAGE inhibition in AD and related pathology.

S.N.	Interventions	Model	Treatment Schedule	Observations	References
1	TTP488 (RAGE antagonist)	Transgenic mice overexpressing *APP/PS1*	Oral treatment with TTP488 starting at 12 months of age	TTP488 treatment ameliorated disease progression dose-dependently. Treatment with TTP488 increased in amyloid burden and reduced inflammatory cytokines	[116]
2	sRAGE-mesenchymal stem cells (MSCs)	Aβ_1__–42_ (5 μL; 200 μM) peptides induced AD model in SD rats	sRAGE-MSCs is transplanted for 4 months	Treatment with sRAGE-MSC decreased apoptotic cells, increased neuron survival and reduced inflammatory cytokines (mRNA of TNF-α, INF-γ and IL-1β) in Aβ_1–42_ administered rats. Transplanted sRAGE-MSCs showed improved survival rate compared to MSCs as evidenced by elevated mRNA levels of CD44, CD90 and CD117 for sRAGE-MSCs.	[133]
3	Hesperidin (20, 40 and 80 mg/kg)	AD like pathology in *APP/PS1* mice	Treatment for 90 days	Treatment with Hesperidin inhibited the increased RAGE expression, the increased phosphorylation of IκBα, terminated nuclear translocation of NF-κB/p65 in the cortex of APP/PS1 mice. Hesperidin reduced oxidative stress (HO-1, SOD, CAT and GSH-Px) and inflammation (TNF-α, CRP, MCP-1 and NF-κB) in cerebral cortex of *APP/PS1 mice.* Treatment with Hesperidin restored learning and memory dysfunction in APP/PS1 mice as evident by decreased escape latency and increased staying in the target quadrant in MWM trial.	[118]
4	Linguizhugan (2.4, 4.8, or 1.2 g/kg)	Aβ-induced (10 µg) AD model in SD rats	Linguizhugan treatment for 25 days	Linguizhugan downregulated the reactive expression levels of RAGE, reduced TNF-α, IL-1β, IL-6, Aβ_1–42_ as well as inhibit MAPK and NF-κB signaling. Linguizhugan ameliorated Aβ-induced spatial learning and memory deficits in MWM trials and improves brain neuronal damage as evident by increased number of neurons in H&E staining.	[117]
5	RAGE specific inhibitor (FPS-ZM1, 1 mg/kg/d, I.P.)	Male APP^sw^/^0^ mice (15 to 17 months old) overexpressing human APP	For 2 months starting at 8 or 15 months of age	FPS-ZM1 bind exclusively to RAGE and inhibited RAGE-driven influx of circulating Aβ_40_ and Aβ_42_ into the brain. Treatment with FPS-ZM1 decrease activity of β-secretase activity, Aβ production and inhibited activation of microglia and the neuroinflammatory mediators (TNF-α, IL-1β, IL-6, and CCL2).	[115]
6	DNMSR (dominant-negative form of RAGE lacking RAGE signaling targeted to microglia)	AD mouse model carrying human mutation of APP (mhAPP) expressing human Aβ	-	Inhibition of microglial RAGE prevented synaptic and behavioural deficits and lowered the activation of stress related kinase (p38MAPK and JNK). Blocking of microglial RAGE signaling prevents entorhinal cortex (EC) synaptic impairment at several stages of neurodegeneration in mhAPP mice.	[134]
7	Pentamidine (0.05 μg/mL) (S100β inhibitor)	Aβ-induced (10 μg/mL) AD in C57BL/6J mice	Per day	Pentamidine treatment reduced the expression of GFAP, S100B, and RAGE protein. Treatment with Pentamidine reduces neuroinflammation (NF-ĸB, IL-1β) and exerted neuroprotection in CA1 pyramidal neurons.	[113]
8	Matrine (10 and 50 μM)	APP/PS1 transgenic mice model		Treatment with Matrine inhibited Aβ_42_-induced cytotoxicity and repress the Aβ/RAGE signaling axis in vitro in SH-SY5Y cells. Matrine treatment downregulated expression of pro-inflammatory mediators (NF-ĸB, IL-1β, and TNF-α), reduced Aβ deposition and ameliorated the memory impairment of AD transgenic mice.	[114]
9	PF-04494700 (10 or 20 mg) (oral RAGE inhibitor)	Subjects with mild-to-moderate dementia of AD type meeting NINCDS-ADRDA criteria	10 week randomized, double-blind, placebo-controlled trial with 2 doses of PF-04494700 (10 mg, after a 6-day loading dose of 30 mg/d); and PF-04494700 (20 mg, after a loading dose of 60 mg/d);	PF-04494700 treatment was safe and well-tolerated in a subject. PF-04494700 treatment exhibited no consistent or clinical effect on plasma levels of Aβ, inflammatory biomarkers (IL-6, IL-1β and TGF-β-1), or secondary cognitive or functional outcomes in this human trial.	[135]
10	PF-04494700 (RAGE inhibitor)	Double-blind, placebo-controlled trial at 40 several centre, subjects assessed with AD assessment scale-cognitive-subscale	Treatment for 18 months using 2 doses of PF-04494700 60 mg/day for 6 days, then 20 mg daily and 15 mg/day for 6 days, then 5 mg daily	High dose of PF-04494700 (20 mg/d) enhanced the adverse effects and cognitive deficits whereas low dose of PF-04494700 (5 mg/d) exhibited a good safety profile.	[136]

AD, Alzheimer’s disease AD; HMGB1, High mobility group box 1; RAGE, Receptor for advanced glycation end products; TLR4, Toll-like receptor 4; Aβ, Amyloid beta; APP, Amyloid precursor protein; PS1, Presenilin 1; MSCs, Mesenchymal stem cells MSCs; IL, Interleukin; IBA1, ionized calcium-binding adapter molecule 1; NINCDS-ADRDA, National institute of neurological and communicative diseases and stroke/Alzheimer’s disease and related disorders association; NF-κB, Nuclear factor κ light chain enhancer of activated B cells; CA, Cornu ammonis; CAT, Catalase; SOD, Superoxide dismutase; TNF-α, Tumor necrosis factor-α, TGF-β1, Transforming growth factor-β1; GFAP, Glial fibrillary acidic protein; MAPK, Mitogen-activated protein kinase; IL, Interleukin; iNOS, Inducible nitric oxide synthase; MWM, Morris water maze.

**Table 3:** Summaries of pre-clinical studies investigating TLR4 inhibition in AD-like pathology.

S.N.	Interventions	Model	Treatment Schedule	Observations	References
1	Monophosphoryl lipid A, LPS-derived TLR4 agonist (MPL, 50 μg, I.P.)	AD like pathology in APP_swe_/PS1 mice	Administered once a week for 12 weeks	TLR4 stimulation with MPL ameliorate AD-like pathology as well as stimulates the phagocytic capacity of innate immune cells. Treatment with MPL reduced Aβ load (number and size of Aβ deposit) in the brain of APP_swe_/PS1 mice and ameliorated cognitive decline as assessed by T-maze.	[120]
2	MPL (1 μg/5 μL/rat)	Aβ_1__–42_-induced (0.075 μg/hour, I.C.V. for 2 weeks) AD related cognitive decline in male Wistar rats	MPL treatment for 24 days (8 injections alternate 3 days)	Early slight activation of microglia by MPL protect synaptic function and improve learning and memory performance. MPL treatment induced dose-dependent release of TNF-α and CCL-3 from BV-2 cells. Treatment with MPL upregulated hippocampal expression of IL-10 and TGF-1β, and arginase 1.	[126]
3	Gx-50 (1 mg/kg)	*APP* transgenic model of AD	Gx-50 administered daily for 2 months at 5 months of age	Gx-50 treatment inhibited TLR4-mediated inflammatory (reduced both TLR4 mRNA and TLR4 proteins) signal cascade in microglial cells and in APP-transgenic mice. Gx-50 treatment inhibited the expression of TNF-α, IL-1β, NO, PGE2, iNOS and COX-2 in Aβ treated rat microglia.	[121]
4	Hesperetin (50 mg)	Aβ_1__–42_-induced (5 μL/5min/mouse) AD model in (C57BL/6N, WT) mouse	Hesperetin (50 mg) treatment for 6 weeks	Hesperetin regulates AD-like pathology by regulating APP, BACE-1, and Aβ. Hesperetin treatment conferred neuroprotection via inhibition of oxidative stress (decrease LPO, ROS and increase Nrf2 and HO-1) neuroinflammation (decreased TLR4, p-NF-κB, TNF-α, and IL-1β), apoptotic cell death (decreased Caspase-3 and PARP-1) and cognitive consolidation (MWM and Y-maze).	[123]
5	MG53 (2 mg/kg)	LPS-induced (0.25 mg/kg, I.P. once a day for 1 week) neuroinflammation and neurotoxicity (in vitro and in vivo) in male C57BL/6 mice.	MG53 (once a day for 2 weeks) was intravenously administrated through tail vein one week before LPS injection.	In the hippocampus of LPS treated mice, MG53 treatment inhibited LPS-induced neuroinflammation in vivo (decreases IL-1β, IL-6, TLR4, p-IKBα and p-NF-κB) via inhibiting TLR4/NF-κB signaling. Pre-treatment with MG53 ameliorated LPS induced memory deficits as evident by shorter escape latency, greater portion of time spent in the target quadrant in MWM trail.	[124]
6	Resveratrol	In vitro study (RAW 264.7 cells stimulated with 10 ng/mL LPS, BV-2 cells 100 ng/mL LPS, and Ba/F3 cells with 50 ng/mL LPS) In vivo study in Aβ APP/PS1 transgenic mice	Orally administered for 15 weeks	50 μM resveratrol treatment inhibited cytokine secretion, NF-κB and STAT1/3 signaling LPS-stimulated BV-2 and RAW 264.7 cells. Resveratrol acted upstream in the activation signaling via interfering with TLR4 oligomerization upon TLR4 stimulation. Resveratrol treatment reduced the number of activated microglial cells surrounding amyloid plaques in APP/PS1 mice.	[125]
7	Baicalin (BAI) (103 mg/kg administered intragastrically)	APP/PS1 transgenic mice	Treated with BAI once a day for 33 days	Treatment with BAI ameliorated learning and memory deficits evident by MWM and PAT and prevented neuronal apoptosis (decreased CASP3 protein) in APP/PS1 mice. BAI suppressed microglial activation and pro-inflammatory cytokine levels (mRNA levels of IL-1β, IL-18, and iNOS), inhibited activation of NLRP3 inflammasome and the TLR4/NF-κB signaling axis but did not decrease Aβ deposition in APP/PS1 mice.	[122]

AD, Alzheimer’s disease AD; TLR4, Toll-like receptor 4; Aβ, Amyloid beta; APP, Amyloid precursor protein; PS1, Presenilin 1; MSCs, Mesenchymal stem cells MSCs; IL, Interleukin; IBA1, ionized calcium-binding adapter molecule; NF-κB, Nuclear factor κ light chain enhancer of activated B cells; LPS, Lipopolysaccharide; TNF-α, Tumor necrosis factor-α, LPO, Lipid peroxides; TGF-β1, Transforming growth factor-β1; NLRP3, Nod-like receptor protein 3; ROS, Reactive oxygen species; IL, Interleukin; iNOS, Inducible nitric oxide synthase; MWM, Morris water maze; STAT1/3, Signal transducer and activator of transcription 1/3.

## Abbreviations

AD, Alzheimer’s disease AD; HMGB1, High mobility group box 1; RAGE, Receptor for advanced glycation end products; TLRs, Toll-like receptors; DAMP, Damage-associated molecular patterns; APP, Amyloid precursor protein; NFTs, Neurofibrillary tangles; PHFs, Paired helical filaments; AGER, Advanced glycation end product specific receptor; PRRs, Pathogen recognition receptors; AHN, Adult hippocampal neurogenesis; NPCs, Neural progenitor cells; BBB, Blood-brain barrier; CNS, Central nervous system; PTMs, Post-translational modifications; MARCKS, Myristoylated alanine-rich C-kinase substrate; TREM2, Triggering receptor expressed on myeloid cells 2; DCs, Dendritic cells; GFAP, Glial fibrillary acidic protein; IL, Interleukin; IBA1, ionized calcium-binding adapter molecule 1; iNOS, Inducible nitric oxide synthase; NF-κB, Nuclear factor κ light chain enhancer of activated B cells; TNF-α, Tumor necrosis factor-α, LPS, Lipopolysaccharide; CD, Cluster of differentiation; STAT3, Signal transducer and activator of transcription 3; NLRP3, Nod-like receptor protein 3; ICV, Intracerebroventricular; CSF, Cerebrospinal fluid; MCI, Mild cognitive impairment; LOAD, late-onset AD.
